# In Vivo Antibacterial and Biochemical Effects of *Moringa oleifera* Leaf Extract in a Rat Model of Multidrug-Resistant *Salmonella* Typhimurium

**DOI:** 10.1155/sci5/5377172

**Published:** 2025-08-08

**Authors:** Megane Nkemendong Nkamkeu, Woquan Sama Luma, Laupy Anne Awah, Thierry Roland Kang, Methodius Shinyuy Lahngong, Moses Njutain Ngemenya

**Affiliations:** ^1^Department of Biomedical Sciences, Faculty of Health Sciences, University of Buea, P.O. Box 63, Buea, South West Region, Cameroon; ^2^Department of Biochemistry and Molecular Biology, Faculty of Science, University of Buea, P.O. Box 63, Buea, South West Region, Cameroon; ^3^Department of Pharmacy, Faculty of Medicine, Pharmacognosy Laboratory, Center of Interdisciplinary Research on Medicine, University of Liège, Liège 4000, Belgium; ^4^Department of Medical Laboratory Science, Faculty of Health Sciences, University of Buea, P.O. Box 63, Buea, South West Region, Cameroon

**Keywords:** antibacterial, *Moringa oleifera*, multidrug resistance, *Salmonella* Typhimurium

## Abstract

The burden of *Salmonella* infections remains high largely due to emergence of antibiotic-resistant strains. *Moringa oleifera* used in traditional medicine to treat bacterial infection has shown considerable antibacterial activity in several in vitro studies but has not been extensively investigated in vivo. The aim of this study was to assess the antibacterial activity of this plant in a rat model of a multidrug-resistant (MDR) *Salmonella* strain as a potential alternative treatment. A MDR strain of *Salmonella* Typhimurium was detected using antibiotic susceptibility. Then a methanol extract of *M. oleifera* leaves was prepared, and the minimum inhibitory concentration (MIC) against the MDR strain was determined by microdilution. Rats were infected with the MDR *S.* Typhimurium, and the extract (125–500 mg/kg of body weight) was administered orally for 10 days. The fecal load of the bacterial colonies was determined by the plate count method. On day 11, the rats were sacrificed, blood was collected, and biochemical effects on liver and renal functions were assessed in serum. The extract showed high activity against the MDR *S.* Typhimurium in vitro with a MIC of 2 mg/mL. There was a significant decrease in fecal colonies at all treatment doses compared to the negative control group (*p* < 0.01), indicating high efficacy of the extract. Optimal in vivo activity of 100% inhibition was observed at 500 mg/kg on day 10. There were no significant changes in biochemical parameters (*p* < 0.05), and no mortality was recorded indicating lack of adverse effects. The high in vivo anti-*Salmonella* activity of the methanol extract of *M. oleifera* coupled with no adverse effects supports its use in traditional medicine. Hence, it is a potential alternative treatment for resistant *Salmonella* infections. *M. oleifera* should be further investigated in vitro and in vivo against other resistant bacteria.

## 1. Introduction

The global morbidity of *Salmonella* infections is persistently high and is associated with considerable mortality [1, 2]. The infections are classified as both typhoidal and nontyphoidal caused by several serovars. Typhoid fever is caused by *S*. Typhi while paratyphoid fever is caused by *S.* Paratyphi A, B, C, and *S.* Sendai infections in humans only with severe disease and high mortality. Nontyphoidal infection is caused by *S.* Enteritidis, *S.* Typhimurium, *S.* Newport, and *S.* Heidelberg, in both humans and animals, and is less severe except in susceptible and compromised persons. Both are transmitted mainly through contaminated food and water [3]. Morbidity and mortality are on the rise due to increasing resistance to the treatment antibiotics. Presently, emergence of resistance has given rise to multidrug-resistant (MDR), extensively resistant, and pan-drug resistant strains of *Salmonella* [4, 5]. Highly resistant invasive nontyphoidal *Salmonella* has been reported in Africa [6, 7]. Fluoroquinolones, ceftriaxone, and azithromycin have been used as alternative antibiotics to treat *Salmonella* infections following emergence of resistance to the initially used antibiotics (penicillins, sulfamethoxazole/trimethoprim, and chloramphenicol), [8]. However, resistance has also emerged against these alternatives leading to the use of carbapenems and colistin against MDR strains. Even then, genes for resistance to these antibiotics have been found [8]. Due to the high magnitude of resistance in *Salmonella*, the bacterium is listed as a priority 2 pathogen indicating a high urgency for new antibiotics to treat its infections [8]. The emergence of resistance makes the search for alternative anti-*Salmonella* treatments an imperative. Approaches to counter MDR *Salmonella* include use of combination therapy, efficacious vaccines, and alternative antibacterials such as probiotics and natural products from medicinal plants [1, 9].

Medicinal plants have shown great potential as a source of new antibacterials and alternatives or complementary antibacterials [9], and some plant products have been approved for clinical use [10]. Studies have demonstrated high antibacterial activity of plant natural products against MDR bacterial strains alone and in combination with antibiotics, with a reduced minimum inhibitory concentration (MIC) of antibiotics, antibiofilm activity, and inhibition of efflux pumps [9].

This study focused on *Moringa oleifera* (Moringaceae), a tree which grows in tropical areas and has nutritional and medicinal uses. It is used traditionally to improve and boost memory, to treat several ailments including infections, diarrhea, respiratory disease, and several other noninfectious diseases [11]. Studies have demonstrated a wide range of pharmacological activities including anticancer, antidiabetic, plasma lipid-lowering, protective, anti-inflammatory, and antimicrobial properties [11].

Several studies have reported on the in vitro antibacterial activity of *M. oleifera*; most used only the disc diffusion method which gives unreliable results. Weak activity was recorded using disc diffusion for leaf extracts against MDR *Escherichia coli*, *Pseudomonas aeruginosa*, and *Staphylococcus aureus* [12]. Broth microdilution studies of the leaves recorded moderate to high activity of the methanolic extract against *Enterococcus faecalis* [13], the ethanolic extract against *E. coli*, the aqueous extract against *Salmonella* [14], and the methanol extract against *S*. Typhimurium among other bacterial species [15]. Moderate to high activity was recorded for the leaf extract against MDR *Salmonella* in vitro, and no adverse effects were observed in an acute toxicity test in mice [16]. On the other hand, in vivo studies of the antibacterial activity are rare but some related in vivo studies have been reported. Sherein et al. [17] reported significant antibacterial activity in uninfected rabbits administered a feed formulation containing *M. oleifera* dry leaf powder. A significant reduction in fecal bacterial colonies was recorded, but the limitation in this study is that a model of bacterial infection was not used. Another study reported significant healing of excision wounds infected with *P. aeruginosa* and *S. aureus* in a rat model treated with a methanol extract of *M. oleifera* [18]. A recent comprehensive review on *M. oleifera* mentioned only one in vivo study which showed higher cysticidal activity of *M. oleifera* leaf extract in combination with praziquantel in rats compared to control animals [19]. The paucity of data on in vivo studies despite reported high in vitro activity of *M. oleifera* leaf extracts motivated this study which is focused on the in vivo activity of the leaf extract in a model of *Salmonella* infection in Wistar rats.

Furthermore, this study was necessitated by the increasing resistance in *Salmonella* which aggravates the already high morbidity and mortality of *Salmonella* infections; the consequence of this is a negative impact on the quality of life, reduced life expectancy, and a decrease in economic growth in the affected population. The increasing resistance together with the reported high in vitro antibacterial activity against MDR *Salmonella* and the lack of toxicity of the leaf extract of *M. oleifera* in mice constitute the rationale for the leaf extract to be studied in vivo [8, 16]. As justification, a good in vivo activity will qualify the plant as a potential alternative treatment which will reduce the disease burden and improve the health and welfare of the affected population. Therefore, the aim of this study was to assess the in vivo anti-*Salmonella* activity of the methanol extract of *M. oleifera* leaves in a rat model of resistant *S*. Typhimurium as a potential treatment for *Salmonella* infections.

## 2. Materials and Methods

### 2.1. Plant Collection

The leaves of *M. oleifera* were harvested in Limbe, in the South-West Region of Cameroon. A voucher sample was prepared and taken to the Limbe Botanic Garden herbarium where it was authenticated by Mr. Njimba Peter, a botanist, by comparing with herbarium voucher specimens. It was identified as *Moringa oleifera* corresponding to the herbarium voucher specimen number SCA6639.

### 2.2. Preparation of Extract

The leaves were dried under shade for 2 weeks, ground to fine powder using a blender, and weighed. Five hundred grams (500 g) of the powder was macerated by completely submerging in 5 L of methanol for 72 h with occasional stirring. Then the mixture was filtered using Whatman filter paper No. 1 and concentrated to a soft extract using a rotary evaporator (BUCHI Rotavapor R-200, Switzerland) at 40°C. The concentrated extract was kept at room temperature to completely evaporate residual solvent and then weighed and stored at 4°C until used.

### 2.3. Antibiotic Susceptibility of *S.* Typhimurium Isolate

*Salmonella enterica serovar* Typhimurium (*S*. Typhimurium) was isolated from a clinical specimen in Yaoundé Teaching Hospital in Cameroon. The isolate was further confirmed by culture on Salmonella-Shigella (SS) agar (colonies appear colorless with black centers). Susceptibility was determined using the Kirby–Bauer disc diffusion method as previously described [20]. Commercial discs of antibiotics belonging to five chemical classes including ampicillin (10 μg), ciprofloxacin (5 µg), chloramphenicol (10 μg), ceftriaxone (12 μg), and sulfamethoxazole/trimethoprim (10 μg) were tested. These were selected on the basis of their use in the clinical management of *Salmonella* infections. McFarland standard was prepared using barium chloride and sulfuric acid following standard procedure. A suspension of the *Salmonella* was prepared in 0.9% saline and standardized to McFarland 0.5 (approximately 1.5 × 10^8^ CFUs/mL). A hundred microliter (100 μL) of the bacterial suspension was transferred onto freshly prepared Mueller–Hinton agar in a culture plate and spread with a sterile spreader to uniformly distribute the cells on the entire surface of the agar. The antibiotic discs were uniformly placed on the bacterial spread. The plate was incubated inverted at 37°C for 24 h, and the diameters of clear zones of inhibition were measured using a ruler in millimeters. The zones of inhibition of the antibiotics were interpreted based on reference values of the Clinical and Laboratory Standards Institute [21].

### 2.4. Determination of MIC of Extract

The microdilution method was used to determine the MIC of the crude extract as previously described [22] with some modifications. The assay was set up in a 96-well microliter plate in sterile conditions. The extract (20 mg/mL) was dissolved completely in 100 μL of DMSO, and then 900 μL of Mueller–Hinton broth (MHB) was added and vortexed. This stock solution was diluted in MHB to obtain 8 concentrations (0.25, 0.5, 1, 2, 4, 8, 10, and 20 mg/mL). Each solution (100 μL) was put in required wells in duplicate, and 100 μL of bacterial suspension (approximately 5 × 10^6^ CFUs/mL from a 1 in 30 dilution of McFarland 0.5 standard suspension) was added to give final concentrations of 0.125–10 mg/mL.

Wells of gentamicin (50 μg/mL) and bacterial suspension only were included as positive and negative controls, respectively. Optical densities (ODs) of the wells were read at 595 nm and incubated at 37°C for 24 h. After 24 h, the plates were visually observed for inhibition and ODs were read again; the change (∆OD) was determined and percentage inhibition of bacterial growth was calculated using the following formula:

(1) $$\begin{array}{c} \%\text{inhibition}=\frac{∆\text{OD Negative control}-∆\text{OD Test }}{∆\text{OD Negative control}}\times 100, \end{array}$$

where change in ∆OD = OD at 24 h–OD at 0 h. MIC was taken as the lowest concentration which showed > 50% inhibition of bacterial growth.

### 2.5. Animals and Ethical Approval

Wistar rats (36), which were 10 weeks old and whose body weights ranged from 80 to 100 g, were used in this study. The animals were purchased from the Laboratory of Animal Biology and Physiology of the University of Yaounde I. They were initially acclimatized in the animal house for 2 weeks in a well-ventilated, illuminated room (12 h light–dark cycle) and provided food and water *ad libitum* daily. The protocol of the animal experiment was reviewed by the University of Buea Institutional Animal Care and Use Committee, and an ethical clearance (Approval reference: UB-IACUC NO 24/2024) was issued approving the study.

### 2.6. In Vivo Antibacterial Assay

The assay was done as described [23, 24] following guidelines of the Organization for Economic Cooperation and Development (OECD), version 423 [25]. The study adhered to the ARRIVE guidelines for animal research. The experiment was conducted in a well-ventilated quiet room, with 12 h dark–light cycle. The animals were weighed and placed into six experimental groups of 6 animals each with one animal per cage. Animals were grouped by stratified randomization with equal males and females and body weight approximately balanced across the six groups. The groups comprised NC (negative control, infected), test groups (T1, T2, and T3, all infected), PC (positive control, infected), and normal group (uninfected). Rats were fasted overnight prior to infection (no food but water only). All animals (except those of the normal group) were infected orally by gavage with the MDR strain of *S*. Typhimurium at 3 × 10^8^ CFUs/mL (McFarland 1 standard, 10 mL/kg volume) in 0.9% saline. Establishment of infection was determined according to Tala et al. [26]. Briefly, fresh rat stool (0.5 g) was dispersed in 1 mL of 0.9% saline in a sterile tube and centrifuged at 2000 g for 5 min. A 1:10 dilution of the supernatant was made and spread on freshly prepared SS agar. The plate was incubated at 37 °C for 24 h and observed for *Salmonella* colonies which were then counted visually. Rectal temperature was monitored by inserting a thermometer bulb, and the stool was observed for diarrhea. Fecal *Salmonella* load increased over three consecutive days with increase in rectal temperature, and diarrhea was observed on day three, all of which confirmed establishment of infection.

Doses of the extractwere deduced from the MIC of the extract (2 mg/mL equivalent to 2000 mg/kg), on the MDR strain of *S.* Typhimurium The doses were chosen to fall within the lower range of OECD recommended doses. Three groups of animals received extract doses of 125, 250, and 500 mg/kg body weight, respectively, by oral gavage using a 20 gauge needle in a maximum volume of 10 mL/kg. Ciprofloxacin (8 mg/kg) was given to PC while NC and NG were given distilled water. Food and water were provided throughout the treatment period. The animals were treated daily for 10 days. Animal bacterial load was determined in feces on days 1, 5, and 10 of treatment as above.

### 2.7. Biochemical Analyses

At the end of the treatment period, the animals were fasted overnight and then anesthetized with ketamine/xylazine (90/10 mg/kg), depending on body weight. Blood was collected by retro-orbital bleeding in an Eppendorf tube and centrifuged at 2000 g for 5 min. The serum was used to test for liver enzymes (aspartate aminotransferase [AST] and alanine aminotransferase [ALT]) and kidney function (urea and creatinine) using diagnostic test kits (Chronolab, Switzerland), according to the manufacturer's instructions.

### 2.8. Data and Statistical Analysis

Data on biochemical analysis were expressed as mean ± standard error of the mean. Data were analyzed using Statistical Package for Social Sciences and GraphPad prism Version 8.4.2. Tukey's multiple comparison test was used to compare fecal loads (total colony counts) of experimental groups, and a *p* < 0.05 was considered statistically significant. The in vivo biochemical data obtained were expressed as mean ± standard deviation and analyzed using one-way analysis of variance (ANOVA).

## 3. Results

### 3.1. Antibiotic Susceptibility and MIC of Extract

The *S.* Typhimuruim strain showed resistance to 5 classes of antibiotics (penicillins, folate pathway inhibitors, phenicols, fluoroquinolones, and cephems), indicating that it was MDR. Ciprofloxacin showed the highest zone of inhibition of 12 mm (cutoff for resistance: ≤ 15 mm). *S.* Typhimuruim showed moderate resistance to chloramphenicol, sulfamethoxazole/trimethoprim, and ceftriaxone and high resistance to ampicillin with zone of inhibition of 6 mm (cutoff for resistance ≤ 13 mm). The mass of the extract was 27.5 g giving a yield of 5.5%. The microdilution assay for the methanol extract of *M. oleifera* leaves recorded a MIC of 2 mg/mL.

### 3.2. In Vivo Antibacterial Activity of *M. oleifera* Extract

Wistar rats infected with the MDR *S*. Typhimurium strain led to shedding of *Salmonella* in their feces. Establishment of infection was shown by increase in number of colonies in feces of infected animals during the first 3 days, watery feces, frequent defecation, high rectal temperatures (36.6°C and 38.2°C), and loss of appetite. Also, red eyes, body weakness, and piloerection were observed after a few days of infection in some animals.

**Figure 1:**
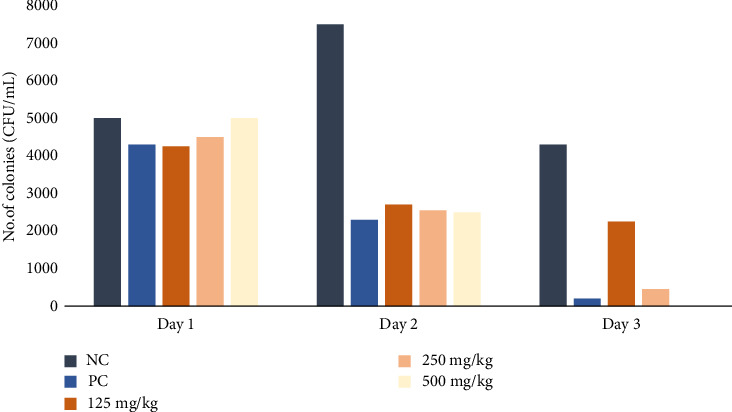
Effect of methanol extract of *Moringa oleifera* leaves on fecal colonies in rats infected with multidrug-resistant *S*. *Typhimurium*. NC = negative control (distilled water); PC = positive control (8 mg/kg ciprofloxacin); extract doses: 125, 250, and 500 mg/kg body weight. *p* < 0.01 on day 10 for negative control compared to treated rats.

The daily oral administration of the extract in infected rats for 10 days induced a marked decrease in the number of viable colonies of *S*. Typhimurium recovered from their feces. On day 5, approximately 20%, 50%, and 80% inhibition of colonies was observed in animals treated with 125, 250, and 500 mg/kg body weight of extract, respectively. On day 10 of treatment, there was 100% inhibition in the feces of animals treated with 500 mg/kg body weight of extract, more than 80% inhibition in animals that received 250 mg/kg body weight, and just 25% inhibition in animals treated with 125 mg/kg as shown in Figure 1. By day 8, the clinical symptoms exhibited by the rats due to the infection subsided compared to the negative control. The negative control (infected and untreated) showed 25% increase in viable *S*. Typhimuruim colonies on day 5 and 25% decrease in viable *S*. Typhimurium colonies on day 10. Meanwhile, the positive control group treated with 8 mg/kg body weight of ciprofloxacin showed a 60% decrease in bacterial load by day 5 of treatment and 90% inhibition of *S.* Typhimuruim colonies on the 10^th^ day. Statistically, there were significant differences between the three test groups and the negative control group with *p* values < 0.01 as shown in Figure 1.

### 3.3. Biochemical Effects on Rats

#### 3.3.1. Serum AST

**Figure 2:**
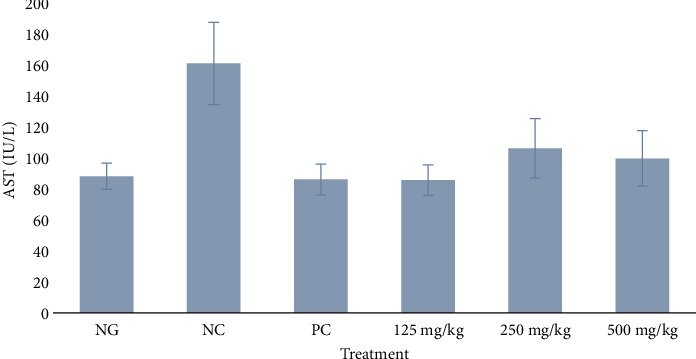
Effect of methanol extract of *Moringa oleifera* leaves on serum aspartate aminotransferase (AST) activity in rats infected with multidrug-resistant *S*. *Typhimurium*. NG = normal group (uninfected, distilled water); NC = negative control (infected, distilled water); PC = positive control (8 mg/kg ciprofloxacin); extract doses: 125, 250, and 500 mg/kg body weight. Significant difference between the test groups and negative control (NC), *p* < 0.0001.

The NC group had the highest mean AST level than all the other groups. There was no significant difference in AST level between the test groups (125, 250, and 500 mg/kg) and the normal group (*p* > 0.05), but there was a significant difference between the test groups and the negative control (*p* < 0.0001). The data also showed a significant difference between the negative control (infected and untreated) and the normal group (uninfected and untreated) with *p* < 0.0001, as shown in Figure 2.

#### 3.3.2. Serum ALT

**Figure 3:**
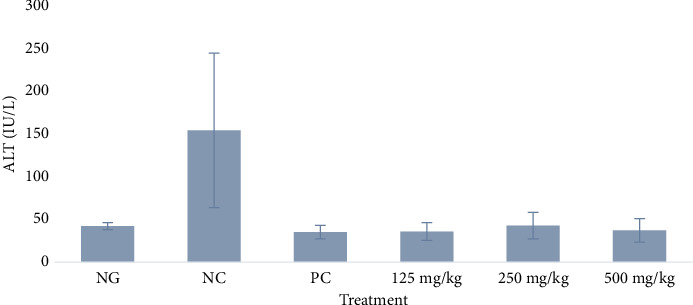
Effect of methanol extract of *Moringa oleifera* leaves on serum alanine aminotransferase (ALT) activity in rats infected with multidrug-resistant *S*. *Typhimurium*. NG = normal group (uninfected, distilled water); NC = negative control (infected, distilled water); PC = positive control (8 mg/kg ciprofloxacin); extract doses: 125, 250, and 500 mg/kg body weight. Significant difference between the test group and the negative control (NC): *p* = 0.0002.

There was a significant difference in ALT level between the test groups and the negative control (*p* = 0.0002), but no significant difference between the test groups and the normal group (*p* > 0.999). There was a significant difference between the normal group and negative control group (*p* = 0.0025), as shown in Figure 3.

#### 3.3.3. Serum Urea

**Figure 4:**
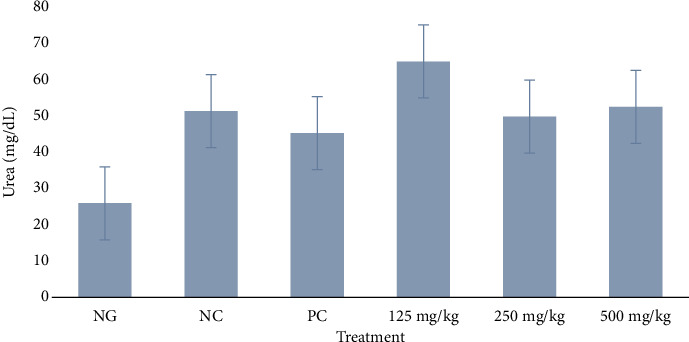
Effect of methanol extract of *Moringa oleifera* leaves on serum urea in rats infected with multidrug-resistant *S*. *Typhimurium*. NG = normal group (uninfected, distilled water); NC = negative control (infected, distilled water); PC = positive control (8 mg/kg ciprofloxacin); extract doses: 125, 250, and 500 mg/kg body weight. No significant difference between treatment groups and negative control, *p* > 0.05.

The 125 mg/kg group had a noticeable higher mean urea level of 64.60 ± 20.89 than all other groups. There was a significant difference between 125 mg/kg and normal group (*p* < 0.05), and no significant difference between 250, 500 mg/kg and the normal group (*p* = 0.3245). There was no significant difference between all the test groups, normal group, and negative control (*p* > 0.05), as shown in Figure 4.

#### 3.3.4. Serum Creatinine

**Figure 5:**
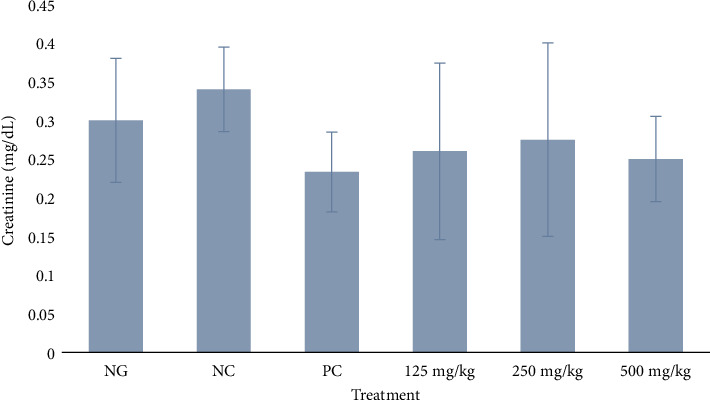
Effect of methanol extract of *Moringa oleifera* leaves on serum creatinine in rats infected with multidrug-resistant *S*. *Typhimurium*. NG = normal group (uninfected, distilled water); NC = negative control (infected, distilled water); PC = positive control (8 mg/kg ciprofloxacin); extract doses: 125, 250, and 500 mg/kg body weight. No significant difference in creatinine level between all the groups, *p* = 0.0354.

Creatinine was generally consistent across all the groups with relatively low standard deviations indicating low variability within each of the six groups. There was no significant difference in creatinine level between all the groups (*p* = 0.0354), as shown in Figure 5.

## 4. Discussion

In this study, the antibacterial activity of *M. oleifera* was assessed in a model of S. Typhimurium infection in Wistar rats in an attempt to find an alternative treatment for MDR strains of these bacteria. This study was motivated by the several reports of moderate to high antibacterial activity of *M. oleifera* in vitro, whereas in vivo studies are rare. The methanol extract of *M. oleifera* leaves was administered to three of the five groups of rats infected with *Salmonella* at 125, 250, and 500 mg/kg, respectively; negative and positive control groups were included and the fecal load of *Salmonella* colonies was recorded as described above. The extract showed a significant reduction of bacterial colonies in the feces of the animals, and no adverse effect or mortality was observed. To the best of our knowledge, this is likely the first report of the in vivo antibacterial activity of *M. oleifera* in a rat model of a MDR *S*. Typhimurium infection. These findings demonstrate the potential of *M. oleifera* leaves as an antibacterial treatment of resistant *S*. Typhimurium infections and a source of antibacterial natural products.

The susceptibility test revealed that the *S*. Typhimurium strain was MDR, having showed resistance to five chemical classes of antibiotics including those recommended for first line treatment of *Salmonella* infections. The doses (125–500 mg/kg) used in the study were appropriate because they were determined from the MIC and also within the range prescribed in recommended protocols [25]. A dose-dependent decrease in *S*. Typhimurium colonies in the feces of the rats administered the extract was recorded with total (100%) clearance of the infection on day 10 at the highest dose of 500 mg/kg body weight. Furthermore, there was a significant decrease in fecal colonies at all doses on day 10 compared to the negative control (*p* < 0.01). This finding confirms the recent moderate to high in vitro antibacterial activity recorded for the methanol crude extract of the leaves of this plant against MDR *Salmonella* stains in vitro [16]. This in vivo activity further supports the use of *M. oleifera* leaves in traditional medicine; it also indicates that the plant is a potential alternative treatment for resistant *Salmonella* infections and a likely source of an efficacious antibacterial natural product compound. However, this activity could have been influenced by factors such as the age, sex, and strain of animals among others. Phytochemical screening of the leaves has been extensive, and the secondary metabolites detected in several studies were flavonoids, tannins, saponins, terpenoids, phenols, and alkaloids [16, 27–29]. These phytochemicals are likely responsible for the observed high antibacterial activity.

The effects of the extract on some vital organs based on biochemical parameters revealed no adverse effects. AST and ALT activities were significantly higher in the infected negative control rats than the uninfected normal rats (*p* < 0.05), as shown in Figures 2 and 3. This suggests the infection induced some damage in the liver cells probably due to inflammation. On the other hand, the enzyme activities in treated rats were similar to the normal group but significantly lower compared to the negative control (*p* < 0.0001 and 0.0002, respectively). These findings indicate that firstly, the extract did not cause any direct damage in the liver and secondly it blocked the harmful effect of the infection through inhibition or killing of the bacteria cells. The concentrations of urea for the negative control and treated rats were similar (*p* > 0.05), but only the rats treated with 125 mg/kg of extract had significantly higher urea than the rats in the normal group. Also, creatinine concentrations for all experimental groups were similar to the normal control (*p* > 0.05). The findings on creatinine suggest the extract had a very negligible toxicity in the kidney. This implies that the significantly high urea recorded at the lowest dose of 125 mg/kg was likely produced in the liver where this dose had the least effect against the damage induced by the infection. These findings are similar to those of a recent study of the acute toxicity of the hexane and methanol extracts of the leaves of *M. oleifera* in uninfected mice, except for a significant increase in AST in the mice induced by the methanol extract. However, despite the high AST, no mortality was recorded in this previous study [16].

Reports on in vivo studies of *M. oleifera* are not readily found in the literature, but the toxicity of this plant has been extensively investigated in many other studies. Two previous studies found no adverse toxicity in both acute (2000 mg/kg) and 28 day repeated dose (100–500 mg/kg) tests [30, 31]. But some other studies found a mixed profile of toxicity and safety at higher doses (3000 mg/kg) [32, 33]. The findings on toxicity in this in vivo study show that at low doses (≤ 500 mg/kg), there is a very low risk of toxicity and these findings are consistent with those of other studies conducted at low doses [30, 31].

The strength of this study is the finding of high in vivo antibacterial activity of *M. oleifera* against MDR *Salmonella* which has not been previously reported. However, there are some limitations in this study. Toxicity analysis was limited to the liver and kidney and should be extended to other systems such as the hematopoietic system. Also, histological analysis of the liver and kidney was not done. Furthermore, there is need to determine the phytochemical profile or fingerprinting of the extract to improve reproducibility and to standardize it to a specific marker. Future work will be extended to cover these aspects.

## 5. Conclusion

This is the first report on the in vivo antibacterial activity of *M. oleifera* against MDR *Salmonella*. The high activity and no adverse toxicity support the use of this plant in traditional medicine. The foliage is a potential alternative treatment against MDR bacteria and likely source of an efficacious antibacterial natural product.

## Data Availability

The essential data have been included in the manuscript. Further details can be requested from the corresponding author.

## References

[1] Gut A. M., Vasiljevic T., Yeager T., Donkor O. N. (2018). Salmonella Infection–Prevention and Treatment by Antibiotics and Probiotic Yeasts: A Review. *Microbiology*.

[2] Zizza A., Fallucca A., Guido M., Restivo V., Roveta M., Trucchi C. (2024). Foodborne Infections and *Salmonella*: Current Primary Prevention Tools and Future Perspectives. *Vaccines*.

[3] Lamichhane B., Mawad A. M. M., Saleh M. (2024). Salmonellosis: An Overview of Epidemiology, Pathogenesis, and Innovative Approaches to Mitigate the Antimicrobial Resistant Infections. *Antibiotics*.

[4] Punchihewage-Don A. J., Ranaweera P. N., Parveen S. (2024). Defense Mechanisms of *Salmon*ella Against Antibiotics: A Review. *Frontiers in Antibiotics*.

[5] Abd El-Aziz N. K., Tartor Y. H., Gharieb R. M. A. (2021). Extensive drug-resistant *Salmonella enterica* Isolated From Poultry and Humans: Prevalence and Molecular Determinants Behind the Co-Resistance to Ciprofloxacin and Tigecycline. *Frontiers in Microbiology*.

[6] Feasey N. A., Dougan G., Kingsley R. A., Heyderman R. S., Gordon M. A. (2012). Invasive Non-Typhoidal Salmon*ella* Disease: An Emerging and Neglected Tropical Disease in Africa. *The Lancet*.

[7] Puyvelde S. V., de Block T., Sridhar S. (2023). A Genomic Appraisal of Invasive *Salmonella* typhimurium and Associated Antibiotic Resistance in Sub-Saharan Africa. *Nature Communications*.

[8] Alenazy R. (2022). Antibiotic Resistance in Salmonella: Targeting Multidrug Resistance by Understanding Efflux Pumps, Regulators and the Inhibitors. *Journal of King Saud University Science*.

[9] Elmaidomy A. H., Shady N. H., Abdeljawad K. M. (2022). Antimicrobial Potentials of Natural Products Against Multidrug Resistance Pathogens: A Comprehensive Review. *RSC Advances*.

[10] Quave C. L. (2016). Antibiotics From Nature: Traditional Medicine as a Source of New Solutions for Combating Antimicrobial Resistance. *AMR Control*.

[11] van den Berg J., Kuipers S. (2022). The Antibacterial Action of *Moringa Oleifera*: a Systematic Review. *South African Journal of Botany*.

[12] Eremwanarue O. A., Shittu H. O. (2019). Antimicrobial Activity of *Moringa oleifera* Leaf Extracts on Multiple Drug-Resistant Bacterial Isolates From Urine Samples in Benin City. *Nigerian Journal of Biotechnology*.

[13] Arevalo-Hıjar L., Aguilar-Luis M. A., Caballero-Garcıa S., Gonzales-Soto N., Del Valle- Mendoza J. (2018). Antibacterial and Cytotoxic Effects of *Moringa oleifera* (Moringa) and *Azadirachta indica* (Neem) Methanolic Extracts Against Strains of *Enterococcus faecalis*. *International Journal of Dentistry*.

[14] Ahmed M., Marrez D. A., Abdelmoeen N. M. (2023). Proximate Analysis of *Moringa oleifera* Leaves and the Antimicrobial Activities of Successive Leaf Ethanolic and Aqueous Extracts Compared With Green Chemically Synthesized Ag-NPs and Crude Aqueous Extract Against Some Pathogens. *International Journal of Molecular Sciences*.

[15] El-Sherbiny G. M., Alluqmani A. J., Elsehemy I. A., Kalaba M. H. (2024). Antibacterial, Antioxidant, Cytotoxicity, and Phytochemical Screening of *Moringa oleifera* Leaves. *Scientific Reports*.

[16] Ngemenya M. N., Itoe L. O., Awah L. A., Asongana R., Ndip R. A. (2024). Antibacterial Activity Against Multidrug-Resistant *Salmonella*, Toxicity and Biochemical Effects of *Moringa oleifera* Leaf Extracts in Mice. *Asian Journal of Natural Product Biochemistry*.

[17] Sherein I. A. E. M., El-Badawi A. Y., Omer H. A. A. (2014). Assessment of Antimicrobial Effect of Moringa: *In Vitro* and *In Vivo* Evaluation. *African Journal of Microbiology Research*.

[18] Al-Ghanayem A. A., Alhussaini M. S., Asad M., Joseph B. (2022). Effect of *Moringa oleifera* Leaf Extract on Excision Wound Infections in Rats: Antioxidant, Antimicrobial, and Gene Expression Analysis. *Molecules*.

[19] Pareek A., Pant M., Gupta M. M. (2023). *Moringa Oleifera*: An Updated Comprehensive Review of Its Pharmacological Activities, Ethnomedicinal, Phytopharmaceutical Formulation, Clinical, Phytochemical, and Toxicological Aspects. *International Journal of Molecular Sciences*.

[20] Al-Rifai A., Aqel A., Al-Warhi T., Wabaidur S. M., Al-Othman Z. A., Badjah-Hadj-Ahmed A. Y. (2017). Antibacterial, Antioxidant Activity of Ethanolic Plant Extracts of Some *Convolvulus* Species and Their DART-ToF-MS Profiling. *Evidence-Based Complementary and Alternative Medicine: eCAM*.

[21] Clinical and Laboratory Standards Institute (2018). *Performance Standards for Antimicrobial Susceptibility Testing*.

[22] Ngemenya M. N., Djeukem G. G. R., Nyongbela K. D. (2019). Microbial, Phytochemical, Toxicity Analyses and Antibacterial Activity Against Multidrug Resistant Bacteria of Some Traditional Remedies Sold in Buea Southwest Cameroon. *BMC Complementary and Alternative Medicine*.

[23] Awah L. A., Taïwe G. S., Babiaka S. B., Cho-Ngwa F., Ngemenya M. N. (2024). *In Vivo* Antibacterial Activity of the Methanol Extract of *Voacanga africana* Stapf (Apocynaceae) Stem Bark Against Clinical Multidrug-Resistant *Salmonella* Typhimurium in Wistar Rats. *Scientific African*.

[24] Mbock M. A., Fouatio W. F., Kamkumo R. G. (2020). *In Vitro* and *In Vivo* Anti-Salmonella Properties of Hydroethanolic Extract of *Detarium microcarpum* Guill. & Perr. (Leguminosae) Root Bark and LC-MS-Based Phytochemical Analysis. *Journal of Ethnopharmacology*.

[25] Organization for Economic Cooperation and Development (2002). Test No. 423: Acute Oral Toxicity—Acute Toxic Class Method. *OECD Guidelines for the Testing of Chemicals*.

[26] Tala D. S., Gatsing D., Fodouop S. P. C., Fokunang C., Kengni F., Djimeli M. N. (2015). In Vivo Anti-Salmonella Activity of Aqueous Extract of Euphorbia Prostrata Aiton (Euphorbiaceae) and Its Toxicological Evaluation. *Asian Pacific Journal of Tropical Biomedicine*.

[27] Malhotra S. P. K., Mandal T. K. (2018). Phytochemical Screening and *In Vitro* Antibacterial Activity of *Moringa oleifera* Lam., Leaf Extract. *Archives of Agriculture and Environmental Science*.

[28] Enerijiofi K. E., Akapo F. H., Erhabor J. O., Erhabor J. O. (2021). GC–MS Analysis and Antibacterial Activities of *Moringa oleifera* Leaf Extracts on Selected Clinical Bacterial Isolates. *Bulletin of the National Research Centre*.

[29] Kagia R., Chepkirui C., Walekhwa M. (2021). Antimicrobial Activity of *Moringa oleifera* Leaf Extracts Against Streptococcus Pneumoniae Isolates. *European Journal of Biology and Biotechnology*.

[30] Palomino-Pacheco M., Rojas-Armas J. P., Ortiz-Sánchez J. M., Arroyo-Acevedo J. L., Justil-Guerrero H. J., Martínez-Heredia J. T. (2024). Assessment of Oral Toxicity of *Moringa oleifera* Lam Aqueous Extract and Its Effect on Gout Induced in a Murine Model. *Veterinary World*.

[31] Odumeru E., Njoku C. C., Ijioma S., Kelechi A. (2023). Acute Toxicity, Phytochemicals, and Nutrient Composition of *Moringa oleifera* Leaves, A Plant Used as a Food Supplement in the Tropical Region of Nigeria. *The Journal of Phytopharmacology*.

[32] Asare G. A., Gyan B., Bugyei K. (2012). Toxicity Potentials of the Nutraceutical *Moringa oleifera* at Supra-Supplementation Levels. *Journal of Ethnopharmacology*.

[33] de Barros M. C., Silva A. G. B., Souza T. G. d. S. (2022). Evaluation of Acute Toxicity, 28-Day Repeated Dose Toxicity, and Genotoxicity of *Moringa oleifera* Leaves Infusion and Powder. *Journal of Ethnopharmacology*.

